# Social Encounters and Mood: The Moderating Role of Narcissism

**DOI:** 10.1093/geroni/igab046.961

**Published:** 2021-12-17

**Authors:** Shiyang Zhang, Karen Fingerman

**Affiliations:** The University of Texas at Austin, Austin, Texas, United States

## Abstract

Social contacts may lead to more positive and less negative emotions in late life, yet we know little about how narcissism influences such associations, and whether contacts with close and not-close social partners impact mood differently. This study examined associations between social contacts, narcissism, and mood on the within- and between- person level. Older adults aged 65 + (N = 303) completed ecological momentary assessments in which they reported social contacts and mood every 3 hours for 5 to 6 days. Older adults had higher positive mood after contacting either close or not-close social partners, but only not-close social partners reduced negative mood. Multilevel models found positive associations between average social contacts number and positive mood among people scored lower on narcissism, and positive associations between social contacts and negative mood for those who scored higher on narcissism. Findings suggest the necessity of considering interpersonal differences in interventions targeting well-being.

